# Optimization of a novel lipid extraction process from microalgae

**DOI:** 10.1038/s41598-021-99356-z

**Published:** 2021-10-12

**Authors:** Xiaojie Ren, Chao Wei, Qi Yan, Xin Shan, Mengyun Wu, Xinhe Zhao, Yuanda Song

**Affiliations:** 1 Colin Ratledge Center for Microbial Lipids, School of Agriculture Engineering and Food Science, Shandong University of Technology, Zibo, China; 2 Baolingbao Biology Co., Ltd., Dezhou, China; 3 Department of Spine Surgery, Zibo Central Hospital, Zibo, China; 4 State Key Laboratory of Microbiology, Shandong University, Qingdao, China; 5 Chongqing Academy of Science and Technology, Chongqing, China

**Keywords:** Isolation, separation and purification, Microbiology techniques

## Abstract

Previous study found that the solvent extraction efficiency of lipid in microalgae could be greatly improved by washing algae cells before the second time extraction. Based on the "organic solvents–water–organic solvents" method, this research further studied the effect of four solvent systems (acetone, chloroform/methanol, chloroform/methanol/water, dichloromethane/methanol), two types of water treatment (vortex and ultrasonic), three water treatment time gradient (0 s, 30 s, 120 s) on the lipid extraction at three different microalgae growth stages (3rd day, 5th day, 9th day). The results show that the combination of water treatment type, treatment time and solvent is very important to the efficiency of lipid extraction. The total lipid extracted was generally increased by 10–30% after water treatment. Especially under the condition of 120 s vortex water treatment with dichloromethane/methanol as extraction solvent, the total lipid extracted increased by 61.14%. In addition, microalgae cells at different culture stages had different sensitivity to water treatment. In this study, under the combination of chloroform/methanol/water as extraction solvent and vortex water treatment for 120 s, the highest lipid yield was obtained on the ninth day of cell culture, which accounts 47.88% of the cell dry weight (478 mg/g cell dry weight). The changes of cell morphology and structure after water treatment were studied by scanning electron microscope, and it was found that water treatment could seriously destroy the cell membrane damaged by solvent, thus promoting the release of lipids. This study further optimizes the "solvent–water–solvent" lipid extraction method, which neither produces impurities nor damages the lipid quality, and can reduce the amount of organic solvent applied in the classical lipid extraction method with the same lipid yield, so it has a broad application prospect.

## Introduction

Many microalgae species have been reported to contain large amounts of lipids in the cell and represent promising cell platforms for lipid production^1^. Their lipids can be easily converted into biodiesel by the process of transesterification, therefore signifies their importance as an alternative source of bioenergy in the future^2,3^. TAGs are the long-chain fatty acids (C14–C22), which could be transformed into fatty acid methyl ester. They are the most ideal type of lipids for biodiesel production^2–4^. Most of the researchers all over the world have their focus on genetic manipulation, metabolic pathways of microalgae^5–8^ and upstream process optimization steps to produce lipid-rich cells^9–19^. Although downstream extraction processes usually account for the main part of the cost of bioprocesses, there is limited attention to improving lipid extraction protocols^1,20,21^.

The rate of lipid extraction largely depends on the extraction solvent and cell crushing technology^22–25^. The main extraction methods of lipids include organic solvent extraction, subcritical solvent extraction, supercritical CO_2_ extraction, acid-thermal method, enzyme method, repeated freeze–thaw method, etc.^26^. The extraction of microalgae lipids mostly adopts organic solvent extraction method. This method uses the characteristics of certain solvents can enter the cell through osmosis, and then dissolve and extract the lipids in the cell through molecular diffusion^27^. At present, the commonly used organic solvent extraction method is the chloroform/methanol (2:1) extraction method proposed by Folch in 1959^28^. Bligh and Dyer subsequently provided a lipids extraction method with chloroform/methanol/water (2:2:1), which realized lipid extraction from many kinds of biomass materials^29^. Recently, due to concerns about biosafety, Cequier et al. have proposed a less hazardous dichloromethane/methanol solvent mixture as an alternative to the B. and D. method^30^. However, due to the strong cell wall structure of microalgae, a large amount of lipids is trapped in the cytoplasm by cell walls and cell membranes, and lipids in algal cells are not completely extracted, which affects the final yield of lipids^31–33^. Therefore, the solvent extraction method can be combined with the ultrasonic crushing method, repeated freeze–thaw crushing method and other crushing methods to improve the rate of lipid extraction. The solvent extraction method has the advantages of high lipid yield, high lipid quality, and easy realization of large-scale production. However, because organic solvents are usually toxic and volatile, they are not conducive to environmental protection. Therefore, how to reduce the amount of solvent applied in the microalgae lipids extraction has become a research hotspot in recent years.

The present study aimed to improve the existing organic solvent extraction method of lipid extraction from microalgae. In our previous study^34^, it was found.

that by washing algae cells before the second time extraction improved lipid extraction rate. The added water treatment procedure produced no impurities, and didn’t affect the quality of lipids and also reduced the use of organic solvent. Based on the discovery, this study further compares the lipid extraction yields of different solvent extraction systems added with water treatment step, and compare the effects of different water treatment types (ultrasonic/vortex), different water treatment time gradient (0 s, 30 s, 120 s) on the lipid extraction efficiency at three different microalgae growth stages. Finally, we obtained the optimal “solvent–water–solvent” extraction parameters for microalgae lipid extraction.

## Results and discussion

### Influence of different solvent systems on lipid extraction efficiency in* Phaeodactylum tricornutum*

**Figure 1:**
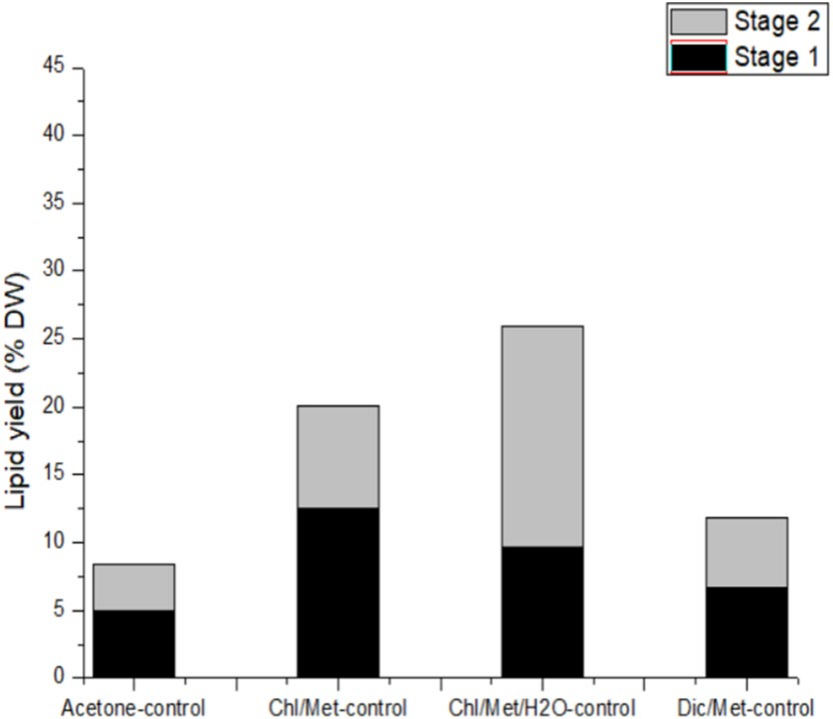
Comparison of extraction efficiency of different extraction solvents (Black bars represent the first-stage lipid extraction yield; grey bars represent the second-stage lipid extraction yield; Chl/Met represents chloroform/methanol extraction method; Chl/Met/H_2_O represents chloroform/methanol/H_2_O extraction method; Dic/Met represents dichloromethane/methanol extraction method. The same below.)

First, the lipid extraction efficiency by different organic solvent systems without adding water treatment step was compared. As shown in Fig. 1, after the first-time extraction by the four organic solvents, there was still a large amount of lipid trapped in the cells that was not completely extracted. The residual lipids accounted for 37.94–62.76% of the total lipids extracted twice (the residual lipids ratio after the first-time extraction equals to lipid amount extracted in the second time divided by the total lipids extracted from the two times extraction). Therefore, it was necessary to obtain the intracellular lipids to the maximum extent after multiple extractions.

In addition, it was found lipid extraction yield was different in different organic solvent systems for *Phaeodactylum tricornutum*. Figure 1 shows the extraction efficiency of four solvent systems, the total lipid extracted is as follows: chloroform/methanol/water (254 mg/g cell dry weight) > chloroform/methanol (201.1 mg/g cell dry weight) > dichloromethane/methanol (118.6 mg/g cell dry weight) > acetone (83.7 mg/g cell dry weight). This is consistent with what was mentioned by Araujo et al.^1^. Jeannotte’s study also showed similar trend in lipid extraction efficiency, in which chloroform/methanol/buffer is the most efficient solvent, followed by chloroform/methanol, hexane/2-propanol and acetone were similar but with less efficiency^35^.

In our result, the lipid extraction efficiency by various solvents was different in the two extractions, in which chloroform/methanol extraction had the highest extraction efficiency in the first-time extraction (124.8 mg/g cell dry weight), however, from the second time extraction, its lipid yield (76.3 mg/g cell dry weight) was much lower than that in chloroform/methanol/water (163.0 mg/g cell dry weight) method. Studies show the rate of lipid extracted largely related to the polarity of the solvent. Ren et al.’s study showed that the extraction efficiency of microalgae lipids was higher with polar solvent compared to nonpolar solvent^34^. Rychecosch et al. as well as Lewis et al. respectively demonstrated that polar and nonpolar solvents mixture successfully extracted more lipids than only nonpolar solvents^23,36^; Kumari et al. also found that the combination of chloroform and methanol exhibited stronger solubility across the entire range of polarity lipids and was more membrane destructive^37^. In this study, chloroform/methanol/water solvent system is more polarity than chloroform/methanol, which may cause in a stronger membrane damage, so when the cells were extracted the second time, its lipid outlet was more, which increased the total lipid extracted.

### Effect of vortex water treatment on the lipid extraction efficiency in Phaeodactylum tricornutum

Previous studies have shown that in *Chlorella protothecoides*, adding water treatment step before the second time solvent extraction step helps to improve the rate of lipid extraction^34^. However, the impact of water treatment on lipid extraction in different algae species still needs to be further verified, and the water treatment type and the parameters of water treatment also need to be optimized. Therefore, in this study, *Phaeodactylum tricornutum* was subjected to further study the effect of different water treatment types (vortex/ultrasonic), different water treatment time (0 s, 30 s, 120 s) combined with the different solvent systems mentioned above in lipid extraction at three different microalgae growth stages.

**Figure 2:**
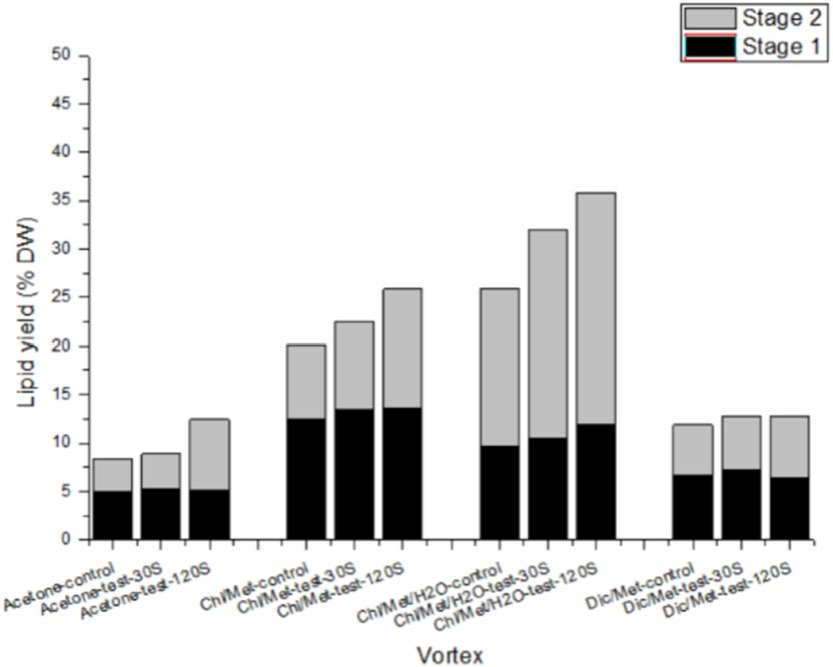
Effect of vortex water treatment on microalgae lipid extraction efficiency.

First, we studied the influence of vortex water treatment on the lipid extraction efficiency. The results (Fig. 2) showed that in each solvent extraction method, before water treatment step, the amount of lipid extracted in the control and test group were equivalent. Then the control group was directly extracted for the second time with the same solvent, while the experimental group was treated with vortex water treatment before the second time solvent extraction. As shown in Fig. 2, the rate of lipid extraction by various solvents was significantly improved after vortex water treatment. The total lipids extracted by the acetone, chloroform/methanol, chloroform/methanol/water, dichloromethane/methanol with a vortex -30 s step were increased by 6.49%, 12.20%, 22.96% and 7.73% respectively compared with the control group. Vortex water treatment effectively improved the extraction efficiency, which may due to the vortex water treated step promote the polarity of the solvent mixture around the cells, and increased lipid effluent by further interfering with cell membrane porosity and integrity. The solvent polarity of chloroform/methanol/water is higher, so chloro- form/methanol/water extraction method has a higher extraction rate of lipids. This is consistent with the effect of lipid extraction rate mentioned earlier.

**Figure 3:**
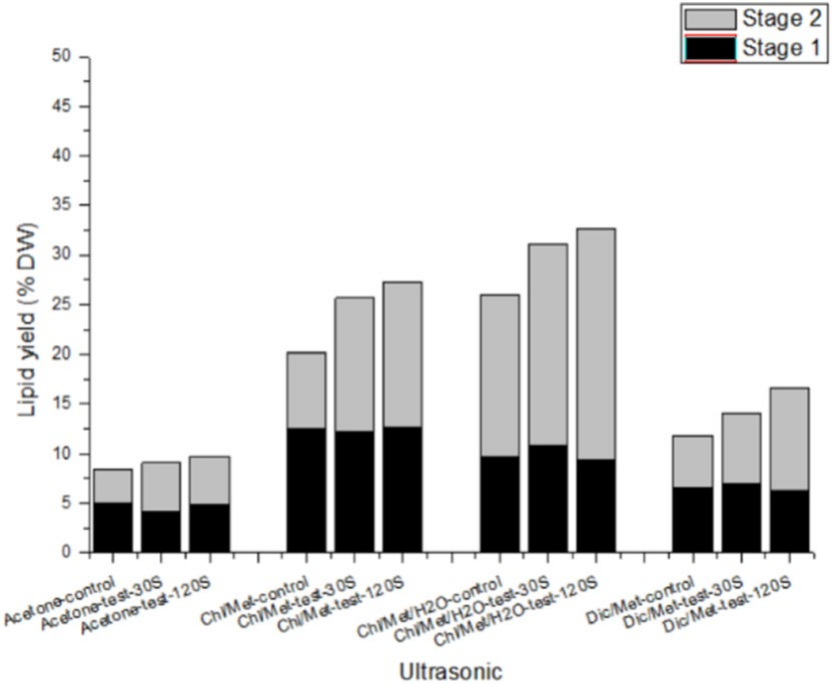
Effect of ultrasonic water treatment on microalgae lipid extraction efficiency.

We further analysed the impact of vortex time on the lipid extraction efficiency. The biomass was treated with vortex water at 0 s, 30 s, 120 s respectively before the second solvent extraction. As can be seen from the Fig. 3, when the time of vortex water treatment was extended, the rate of lipid extraction was increased, which shows a positive correlation between water treatment time and lipid yield.

In the acetone extraction method, the lipid extracted from the second time extraction increased by 6.49% (30 s) and 114.76% (120 s) compared with the control group. This increased percentage value = (lipid obtained from the second extraction in the test group—lipid obtained from the second extraction in the control group)/ lipid obtained from the second extraction in the control group. In the chloroform/methanol extraction method, the lipid obtained in the second extraction increased by 19.31% (30 s) and 60.01% (120 s) respectively compared with the control group. In the chloroform/methanol/water extraction method, the lipid extracted in the second stage increased by 31.34% (30 s) and 47.09% (120 s) respectively compared with the control group. In the dichloromethane/methanol extraction method, the lipid extracted in the second stage increased by 5.35% (30 s) and 22.66% (120 s) respectively compared by the control group. After comprehensive consideration, we observed that using chloroform/methanol/water extraction method combined with vortex water treatment step for 120 s can obtain a relatively high lipid amount (380.0 mg/g cell dry weight), and this combination method has become one of the optimal schemes for lipid extraction of microalgae.

### Effect of ultrasonic water treatment on the lipid extraction efficiency in* Phaeodactylum tricornutum*

The impact of ultrasonic water treatment on the extraction efficiency of microalgae lipid was studied, and the results were shown in Fig. 3. The extraction efficiency of lipids by various solvents was significantly improved after ultrasonic water treatment. The total lipids extracted by the acetone, chloroform/methanol, chloroform/methanol/water, dichloromethane/methanol with an ultrasonic-30 s water treatment step were increased by 8.13%, 27.45%, 19.48% and 18.97% respectively compared with the control group. Among these, chloroform/methanol extraction method has more obvious effect (27.45% promotion) after ultrasonic water treatment, and chloroform/methanol/water extraction method has the highest total lipid extraction yield (310.3 mg/g cell dry weight).

On this basis, we further analysed the impact of ultrasonic time on the rate of lipid extraction. The biomass was treated with 0 s, 30 s, 120 s respectively before the second time solvent extraction, and the results were shown in Fig. 3. With the extension of ultrasonic time, the extracted lipid increased, and the two showed a significant positive correlation. In the acetone extraction method, the lipid extracted in the second stage increased by 42.63% (30 s) and 43.45% (120 s) compared with the control group. In the chloroform/methanol extraction method, the lipid extracted in the second stage increased by 76.05% (30 s) and 91.58% (120 s) respectively compared with the control group. In the chloroform/methanol/water extraction method, the lipid extracted in the second stage increased by 24.17% (30 s) and 42.58% (120 s) respectively compared with the control group. In the dichloromethane/methanol extraction method, the lipid extracted in the second stage increased by 37.14% (30 s) and 98.53% (120 s) respectively compared by the control group. After comprehensive consideration, we observed that using chloroform/methanol/water extraction method combined with ultrasonic water treatment step for 120 s can obtain a relatively high lipid yield (332.6 mg/g cell dry weight).

Therefore, it can be seen from the results of the above four parts that extending the vertex or ultrasonic water treatment time can improve the lipid extraction efficiency. We speculate that after the first step of organic solvent extraction, membrane integrity was damaged, and water is a polar solvent with highly active, after adding water treatment, water molecules may affect the damaged membrane phospholipid bilayer rearrange, thus help to disrupt the cell membrane, makes the second extraction solvent into the intracellular easier, then is advantageous to the lipid’s extraction. Clearly, longer water treatment times will increase the degree of membrane rearrangement and thus facilitate the extraction of organic solvents.

In addition, repeatable experiments from different culture batches were carried out in *Phaeodactylum tricornutum* under the same experiment conditions. They all showed the similar trends as shown in our manuscript. These were provided as supplementary material Figs. S1–S3.

### Comparison of vortex and ultrasonic water treatment on lipid extraction efficiency

The results of vortex and ultrasonic water treatment under the same treatment time were compared (Supplementary material Fig. S4). It showed that when the water treatment is lasted for 30 s, the lipid extraction efficiency was 33.15%, 47.58% and 30.13% higher in ultrasonic water treatment method than that in the vortex water treatment method using acetone, chloroform/methanol and dichloromethane/methanol extraction system respectively. Only in the chloroform/methanol/water extraction system, lipid extraction efficiency was slightly lower in the ultrasonic method than the vortex method (4.48%). Therefore, ultrasonic treatment of biomass is generally more effective than vortex treatment in a short time. After 120 s, the effects of two water treatment are different in various solvents. In chloroform/methanol and dichloromethane/methanol system, lipid extraction efficiency by ultrasonic water treatment was still 19.67% and 61.93% higher than that by vertex water treatment. While, in acetone and chloroform/methanol/water system, vertex water treatment was more effective than ultrasonic method on lipid extraction (the lipid yield was 49.69% and 2.96% higher by vertex than that of ultrasonic treatment method).

Compared with the result at 30 s, the effect of vortex water treatment and ultrasonic water treatment in the acetone extraction system has undergone a significant change in the prolonged water treatment time. The effect of ultrasonic treatment is better than that of vortex water treatment in a short time, while the long-term processing vertex has a higher efficiency than the ultrasonic treatment. In addition, both short-term and long-term water treatment in the chloroform/ methanol/water extraction system show that the vortex water treatment is more effective than the ultrasonic water treatment. In the chloroform/methanol extraction method, although the ultrasonic treatment method surpasses the vortex water treatment in both short-term and long-term treatment, the advantages of ultrasonic water treatment gradually get weakening along with processing time. In short-term treatment, the efficiency of ultrasound is 47.58% higher than that of vortex water treatment, and in long-term treatment, the efficiency of ultrasonic treatment is 19.67% higher than that of vortex water treatment. However, in the dichloromethane/methanol extraction method, after prolonging the treatment time, ultrasonic shows greater advantages than vortex water treatment (30.13% and 61.93% higher at 30 s and 120 s, respectively). Therefore, in summary, water treatment can greatly promote the traditional solvent extraction of microalgae lipids, but different water treatment methods have different effects on the promotion of lipid release when participating in different solvent systems. Therefore, for specific algae species, various combinations of solvents and water treatment methods and parameters need to be optimized specifically. For *Phaeodactylum tricornutum* studied in this paper, chloroform/methanol/water system combined with vortex water treatment for 120 s obtained the highest lipid yield (380.0 mg/g cell dry weight).

### The sensitivity of algal cells at different growth stages to water treatment

The vortex and ultrasonic water treatment effect on lipid extraction of the third, fifth, and ninth days of microalgae growth were compared respectively. We summarized all the experimental results under various extraction conditions (supplied in Supplementary material Table S1), the percentage of total extracted lipid promoted under different parameters were shown in Table 1.

**Table 1:** The percentage of total extracted lipid promoted by water treatment step.

Promoted percentage		Vertex water treatment	Ultrasonic water treatment				
		Day 3	Day 5	Day 9	Day 3	Day 5	Day 9
Acetone	30 s	6.49	0.26	17.71	8.13	14.14	35.15
	120 s	48.59	10.59	5.44	16.33	53.69	60.21
Chloroform/methanol	30 s	12.20	26.93	14.56	27.45	7.42	11.91
	120 s	28.70	29.92	17.17	35.74	27.00	17.73
Chloroform/methanol/H_2_O	30 s	22.96	17.54	13.89	19.48	14.82	9.54
	120 s	38.00	21.80	13.17	25.90	14.22	7.71
Dichloromethane/methanol	30 s	7.73	3.89	11.88	18.97	30.18	20.54
	120 s	7.64	14.97	61.14	40.49	39.10	41.81

Solvent extraction efficiency of lipid in microalgae could be greatly improved by washing algae cells before the second time extraction.

It clearly shows that solvent extraction efficiency of lipid in microalgae could be greatly improved by the water treatment. The percentage of total extracted lipid promoted in the test groups was roughly stable at 10–30%, and the 120 s vortex water treatment had the most obvious effect on the extraction of dichloromethane/methanol, which was 61.14% higher than that of the control group. After comparison of various methods, *Phaeodactylum tricornutum* obtained the highest lipid content under the combined conditions of chloroform/methanol/water as the extraction solvent and vortex water treatment for 30 s on the 9th day of growth, which account for 478.8 mg/g cell dry weight.

Meanwhile, relative literatures using *Phaeodactylum tricornutum* for lipid extraction were also compared^38,39^. In the research of Tommasi et al.^38^, total lipid up to ~ 32% of cell dry weight was obtained using deep eutectic solvents and microwaves to pretreat the biomass during lipid extraction. Which also showed significant higher efficiency compared to organic solvent extractions (5 ~ 10% of cell dry weight). The results in control groups with organic solvent extractions were comparable with our control results. While, the highest lipid extracted were 47.88% of the cell dry weight in our research, which is much more efficient compared with their method and the traditional solvent extraction methods.

### Effects of water treatment on the cell morphology of microalgae

**Figure 4:**
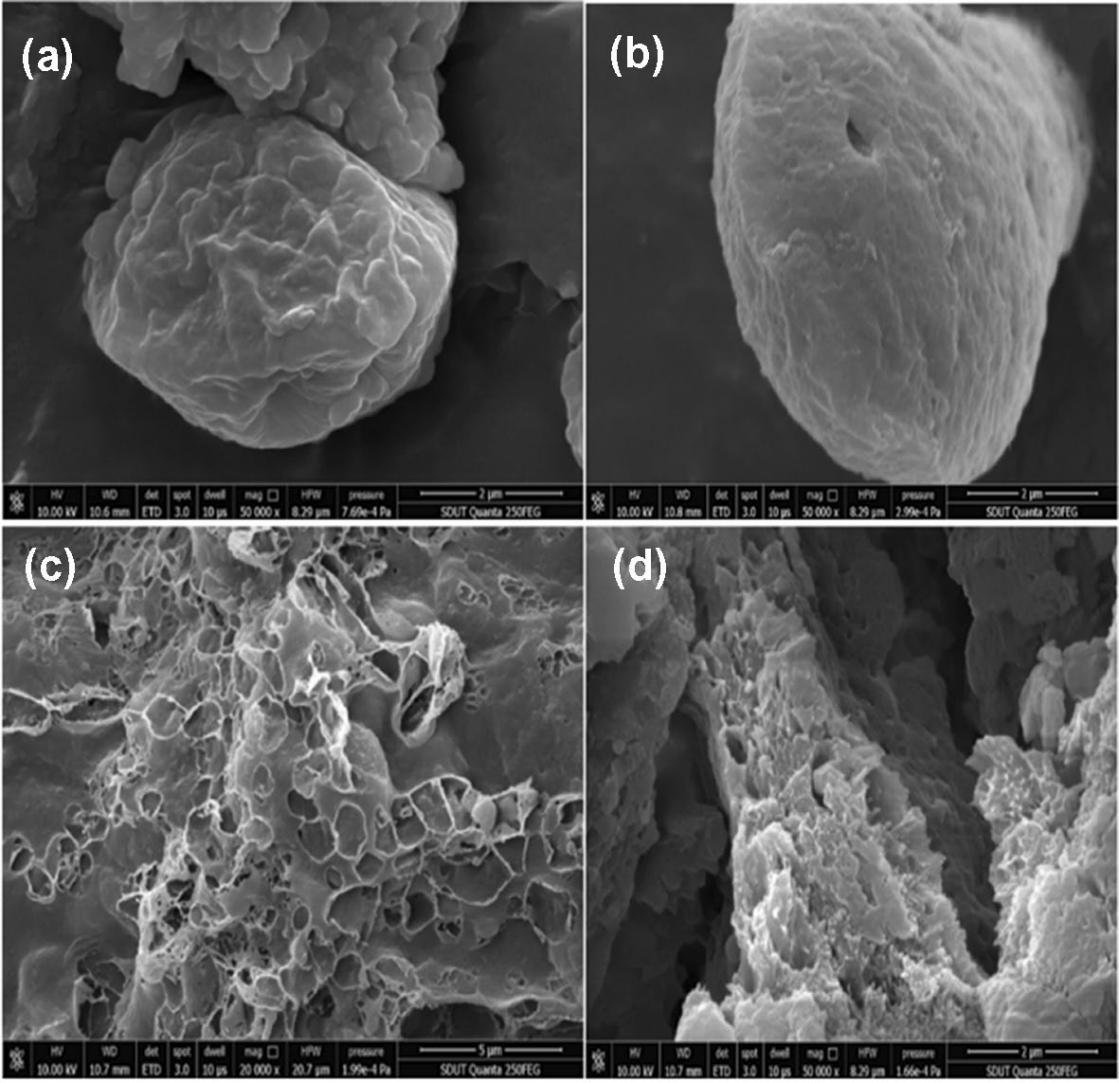
Scanning electron microscopy (SEM) of alga cells in the organic solvent–water- organic solvent extraction process. (**a**) Morphology of algal cells before extraction, (**b**) morphology of algal cells after the first extraction, (**c**) morphology of algal cells after water treatment, (**d**) morphology of algal cells after the second extraction.

We applied this solvent–water-solvent lipid extraction method to *Aurantiochytrium sp*. SW1 with higher lipid content, which also showed that water treatment had a significant promoting effect on lipid extraction rate. We detected the algal cells at four extraction stages: before extraction, after one time extraction, after water treatment, and after the second time extraction with solvent by scanning electron microscopy (SEM). We did SEM image for both *Phaeodactylum tricornutum* and *Aurantiochytrium sp*. SW1, however, only the electron micrograph of *Aurantiochytrium* sp. SW1 can find single cells, which can more clearly reflect the effect of the extraction process on the cell morphology. Therefore, we presented the SEM micrography of *Aurantiochytrium* sp. SW1 in Fig. 4, and the SEM micrography of *Phaeodactylum tricornutum* was provided in the supplementary material (Fig. S5).

The algal cells were freeze-dried before extraction, so it can be seen from the electron microscope that the surface of the cells was still very intact and the cell membrane was not damaged, although there were dried folds and dents (Fig. 4a). However, a small number of holes appeared on the cell surface after the extraction of organic solvent (Fig. 4b). This is because organic solvent can dissolve the phospholipids on the cell membrane, resulting in cell membrane damage. The solvent then penetrates into the cell and extracts the lipid from the cell through molecular diffusion. However, it can be seen from the electron microscope that the solvent does not damage the cell membrane completely, and the lipid extraction is mainly carried out at the place where the cell membrane dissolves through osmosis, which explains that 30–70% of the lipids still remain in the cells after a single solvent extraction. According to the electron microscope Fig. 4c, after the vortex water treatment, the cells were completely disintegrated and lost their complete cellular structure. This may be because pure water has a high activity level of polarity. After the addition of water treatment, the water molecules may affect the rearrangement of the damaged membrane phospholipid bilayer, thus helping to disturb the cell membrane. After a long time of vortex vibration, the cell membrane is destroyed in a large area, so it is more conducive to the extraction of solvent in the next step. Finally, after the secondary solvent extraction, the cells became flocculent (Fig. 4d), so the lipid extraction was more thorough. Therefore, through the observation of scanning electron microscope, we can clearly see the changes in the morphology of algae cells after solvent extraction by water treatment, thus explaining the promoting effect of water treatment on the extraction of microalgae lipids by traditional organic solvent method from the cellular level.

## Conclusion

In this study, we explored a novel lipid extraction method of “organic solvents–water–organic solvents” method. The novel extraction process stimulates the microalgae lipid extraction efficiency and reducing the amount of organic solvent applied in the traditional solvent extraction method. The extraction efficiency of microalgae lipids was significantly improved (10–30%) by adding a vertex or ultrasonic water treatment to the biomass after the first time of solvent extraction. Extending vertex or ultrasonic water treatment time can improve the lipid extraction efficiency. However, the effect of ultrasonic treatment is better than that of vortex water treatment in a short time, while the long-term processing vertex has a higher efficiency than the ultrasonic water treatment. The combination of water treatment type; treatment time and solvent are very important to the efficiency of microalgae lipid extraction. The scanning electron microscope (SEM) show that water treatment could seriously destroy the algae cell membrane damaged by solvent, thus promoting the release of lipids. It is a very efficient and environmentally friendly method that further improves the lipid extraction efficiency.

## Materials and methods

### Strain culture and pre-treatment

*Phaeodactylum tricornutum* (FACHB-2174) was purchased from the Chinese freshwater algae culture collection at the institute of hydrobiology. The culture media used to cultivate *P. tricornutum* was MBM, which contained 40 g/L glucose, 1 g/L glycine, 4 g/L yeast extract, 0.7 g/L KH_2_PO_4_, 0.3 g/L K_2_HPO_4_, 0.3 g/L MgSO_4_·7H_2_O, 3 mg/L FeSO_4_·7H_2_O, 2.86 mg/L H_3_BO_5_, 1.81 mg/L MnCl_2_·4H_2_O, 0.105 mg/L ZnCl_2_, 0.039 mg/L Na_2_MoO_4_·0.2H_2_O, 0.079 mg/L CuSO_4_·5H_2_O and 0.03 mg/L CoCl_2_. The seed culture in the logarithmic growth phase was inoculated (10%, v/v) and then cultivated in the incubator (Infors-HT, Bottmingen, Switzerland) at 28 °C under light^40^. Light was provided by LED lamp configured in the incubator at 60 μmol m^−2^ s^−1^, light to dark photoperiod of 12–-12 h was set to imitate day and night alternation. The rotating speed of the shaker is 150 rpm/min to ensure the ventilation in the cultivation flasks.

*Aurantiochytrium* sp. SW1 was screened from our lab, and the culture medium consist with glucose (60 g/L), yeast extract (2 g/L), monosodium glutamate (8 g/L) and artificial sea salt (6 g/L)^41^. The cultures were kept in the incubator at 28 °C, 200 rpm without light.

The algal cultures (sample) were collected aseptically during different growth periods (3, 5, 9th days). Samples was collected by centrifuging the culture at 8000 rpm for 10 min in refrigerated centrifuge. The obtained biomass was washed with ddH_2_O and centrifuged again. The algae cells were grinded by liquid nitrogen in a mortar and freeze dried in freeze dryer, then the lyophilized algae powder was stored in a refrigerator at − 20 °C for further lipid extraction^42^.

### Lipid extraction method

#### Acetone extraction method^43^

Five samples (10 mg algae powder/sample) were taken and added with 5 mL of acetone solution respectively. The samples were extracted 30 min under ultrasonic in ice water and then centrifuged under 10,000 rpm at 4 °C for 20 min. Transfer the supernatants to liposuction bottles as the first extracts (stage 1) for subsequent analysis. Then one of the five samples was treated as control group and was extracted with 5 ml of acetone repeatedly, the supernatant was transferred to the liposuction bottle as a second extract for subsequent analysis. In the other four test groups, the biomass was added 5 mL ddH_2_O and vortexed for 30 s, 120 s or ultrasonicated for 30 s, 120 s, respectively. After water treatment, the test group samples were centrifuged for 5 min at10,000 rpm, 4 °C, the supernatants were removed, and the cell particles were extracted with 5 ml of acetone again. The supernatants from the second time acetone extraction were transferred to the liposuction bottles as the second extracts (stage 2) of test groups for subsequent analysis.

#### Chloroform/methanol extraction method^28^

The extraction procedure of chloroform/methanol method were the same as the acetone method. Control group was extracted by 7.5 mL chloroform/methanol (volume ratio: 2:1) for two times. For each time, transfer the supernatants to new liposuction bottles for further operation. For the other four test groups, 5 mL distilled water were added after one time of chloroform/methanol extraction, and treated with vertex for 30 s, 120 s or ultrasonic for 30 s, 120 s, respectively. After water treatment, the test group samples were centrifuged for 5 min at 10,000 rpm, 4 °C, the supernatants were removed, and the cell particles were extracted with 7.5 ml of chloroform/methanol mixture (volume ratio: 2:1) again. All the chloroform/methanol extraction superannuants from control and test groups were then stratified by adding 3 mL of 0.9% sodium chloride solution respectively, and centrifuged for 5 min at 4000 rpm. Remove 5 mL of chloroform containing lipid from the lower layer and transfer it to a liposuction bottles as the first (stage 1) or second extract (stage 2) of the control and test groups accordingly for subsequent analysis.

#### Chloroform/methanol/water extraction method^29^

Five samples (10 mg/sample) of dried microalgae were homogenized in chloroform/methanol/water (2.5 mL/5 mL/5 mL). And was subjected to ultrasonic treatment for 20 min in ice water. Add another 2.5 mL of chloroform to the mixture and treat it with ultrasound for 10 min. Then centrifuge the mixture for 5 min at 10,000 rpm. Remove the upper layer of methanol and water mixture to waste tank, and take 5 mL of chloroform containing lipid from the lower layer and transfer it to a liposuction bottle as the first extract (stage 1) for subsequent analysis. The remaining cells precipitate for the second step of lipid extraction.

The control group repeated the first step of lipid extraction. While cell particles in the four test groups after the first step of lipid extraction were added with 5 mL distilled water and were treated with vertex for 30 s, 120 s, or ultrasonic 30 s, 120 s, respectively. The supernatant was removed by centrifugation at 10,000 rpm for 5 min. The extraction of the remaining cell particles was repeated as the first step (homogenized in chloroform/methanol/water with volume ratio 2.5 mL/5 mL/5 mL. And treated by ultrasonic for 20 min in ice water. Add another 2.5 mL of chloroform to the mixture followed by 10 min ultrasonic treatment. Then centrifuge the mixture for 20 min at 10,000 rpm. The methanol and water mixture in the upper layer was absorbed into the waste liquid tank, and the lower 5 mL chloroform containing lipids was transferred into the liposuction bottles as the second extracts (stage 2) of the test groups for subsequent operation and analysis).

#### Dichloromethane/methanol extraction method^30^

The extraction procedure of dichloromethane/methanol method were the same as the chloroform/methanol method. Extracted samples by 7.5 mL dichloromethane/methanol (volume ratio: 2:1) instead of chloroform/methanol. The control group repeated dichloromethane/methanol extraction for two times, while the test groups added a water treatment step of vertex 30 s, vertex120 s, ultrasonic 30 s, ultrasonic 120 s, respectively after the first-time extraction. All the dichloromethane/methanol extractions from control and test groups were then stratified by adding 3 mL of 0.9% sodium chloride solution respectively, and centrifuged for 5 min at 4000 rpm. 5 mL of dichloromethane containing lipid from lower layer was transferred into liposuction bottles as the first (stage 1) or second extract (stage 2) accordingly for subsequent analysis.

**Figure 5:**
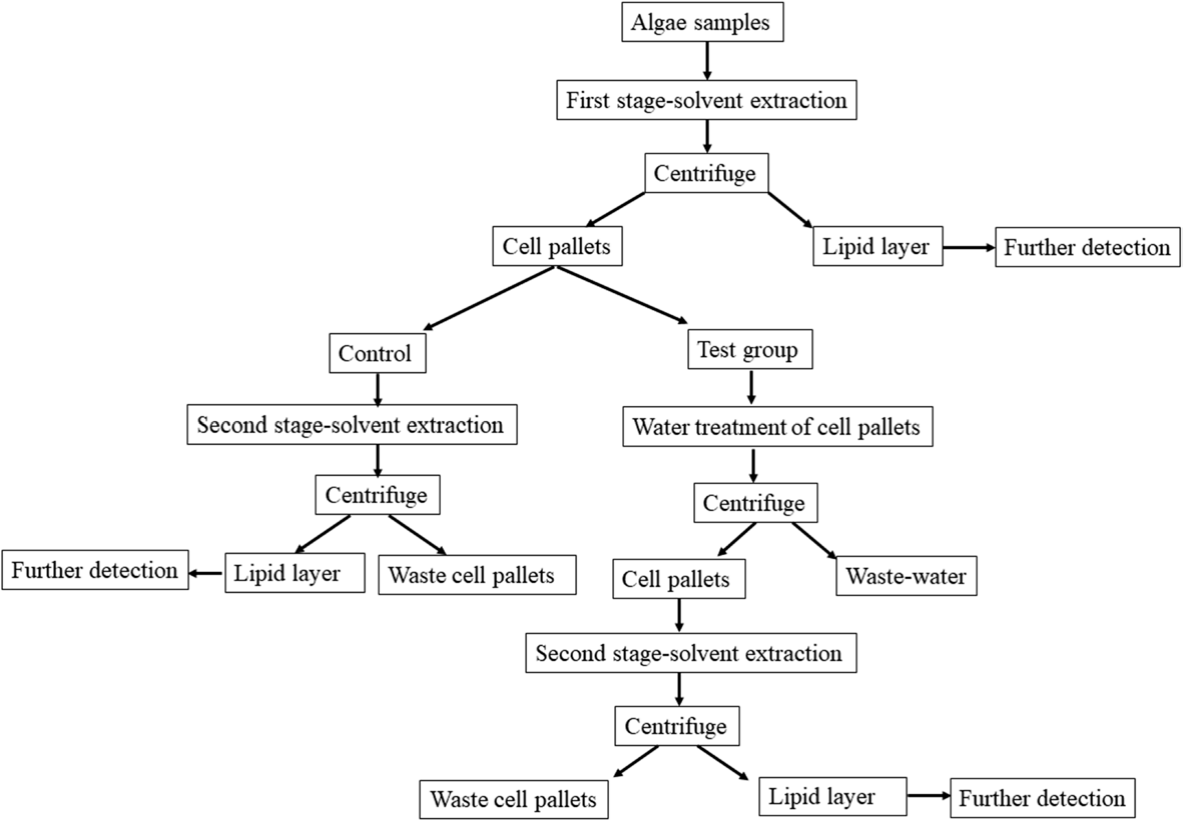
Technology roadmap of the lipid extraction methods.

A technology roadmap was provided to clarify the four lipid extraction methods (Fig. 5).

### Lipid quantification

Extracts of control and test groups from the first and second extraction stages were used for lipid quantification. With reference to the rapid lipid determination method proposed by Drochioiu^41^, lipid content was determined by the turbidity reaction of sulfosalicylic acid. The above organic solvents containing lipid obtained from lipid extraction process was evaporated under nitrogen flow. Then redissolve each sample in 0.2 ml acetone and add 1.8 ml 1.5% sulfosalicylic acid. Shake the sample violently, and then let it stand for 30 min. Then OD440 nm was read by ultraviolet–visible spectroscopy. To generate the standard curve, a reserve solution (2 g/L) was prepared by dissolving a known weight of lipids in acetone, then diluted the reserve solution into a series of standard solutions. The corelation of lipid concentration and OD440 nm was used as the standard curve^34^.

### Observation of cell morphology at different extraction stages by scanning electron microscopy

In order to observe the changes in cell morphology of microalgae after adding water treatment in the process of lipid extraction, scanning electron microscopy was used. Select the extraction method with the largest increase in lipid extraction rate. A few unextracted alga samples after lyophilization were used as sample 1, the alga cells samples after one solvent extraction were used as sample 2, the alga cells samples after water treatment were used as sample 3, and the alga cells after the second solvent extraction step used as sample 4. Lyophilize the above 4 samples (− 80 °C for 48 h) for further cell morphology analysis. Stick clean silicon wafers with a small amount of sample, it is advisable that the cell pellets are dense but not piled together. Place the above samples in 1–2% glutaraldehyde phosphate buffer (pH 7.2) and fix them overnight in a refrigerator at 40 °C. The next day, the samples were rinsed with the same buffer of 0.15% and dehydrated with 40%, 70%, 90% and 100% ethanol for 15 min each time. After dehydration, ethanol was replaced with amyl acetate. In the critical point dryer, the samples were immersed in liquid carbon dioxide and heated to the critical point temperature (31.4 °C, 72.8 atmospheric pressure) above, and then vaporized for drying. Finally, Finally, the sample is put into a vacuum coating machine. Gold is sprayed on the sample, and the sample is taken out and observed under a scanning electron microscope (Quanta 250-field emission environment scanning electron microscope).

## Supplementary Information

Supplementary Information.
