# Supplementary material for: Optimization of a novel lipid extraction process from microalgae
Source: Sci Rep. 2021 Oct 12;11:20221. doi: 10.1038/s41598-021-99356-z (PMC8511141; doi:10.1038/s41598-021-99356-z)
Supplement: Supplementary file 1 — Supplementary Information. [file 41598_2021_99356_MOESM1_ESM.docx]

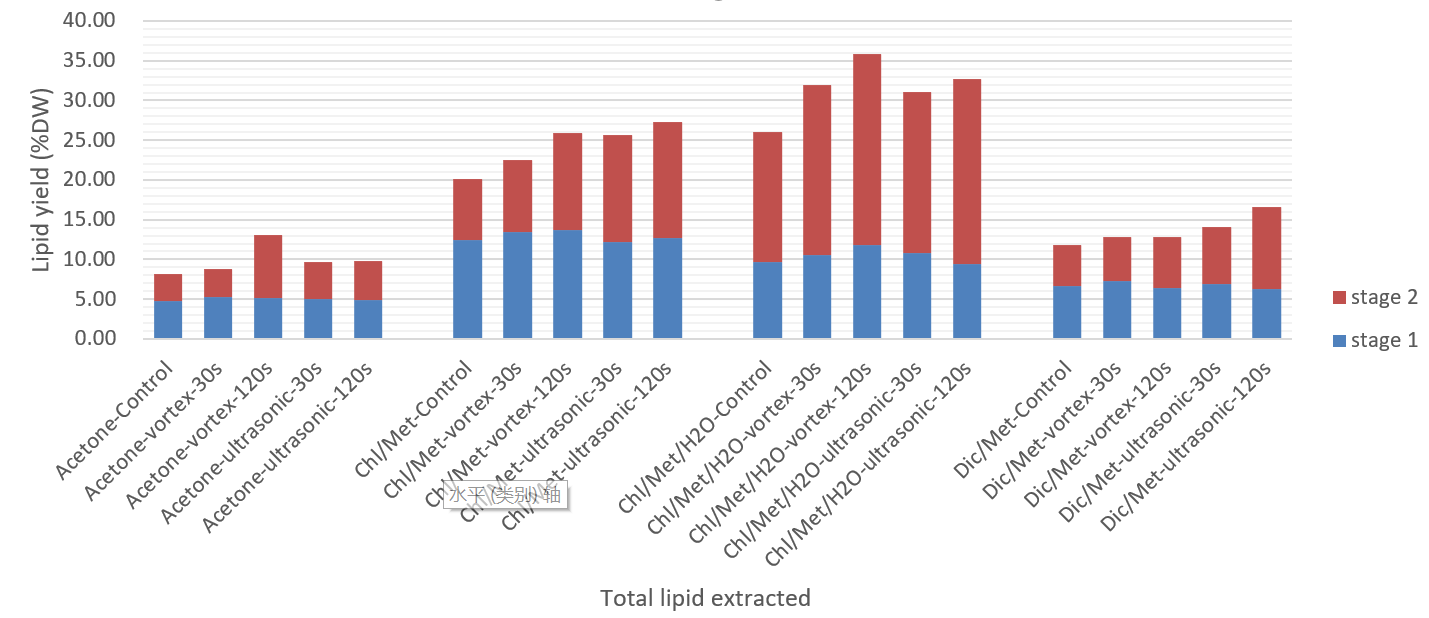


**Fig. S1 Replication-1 in *Phaeodactylum tricornutum***


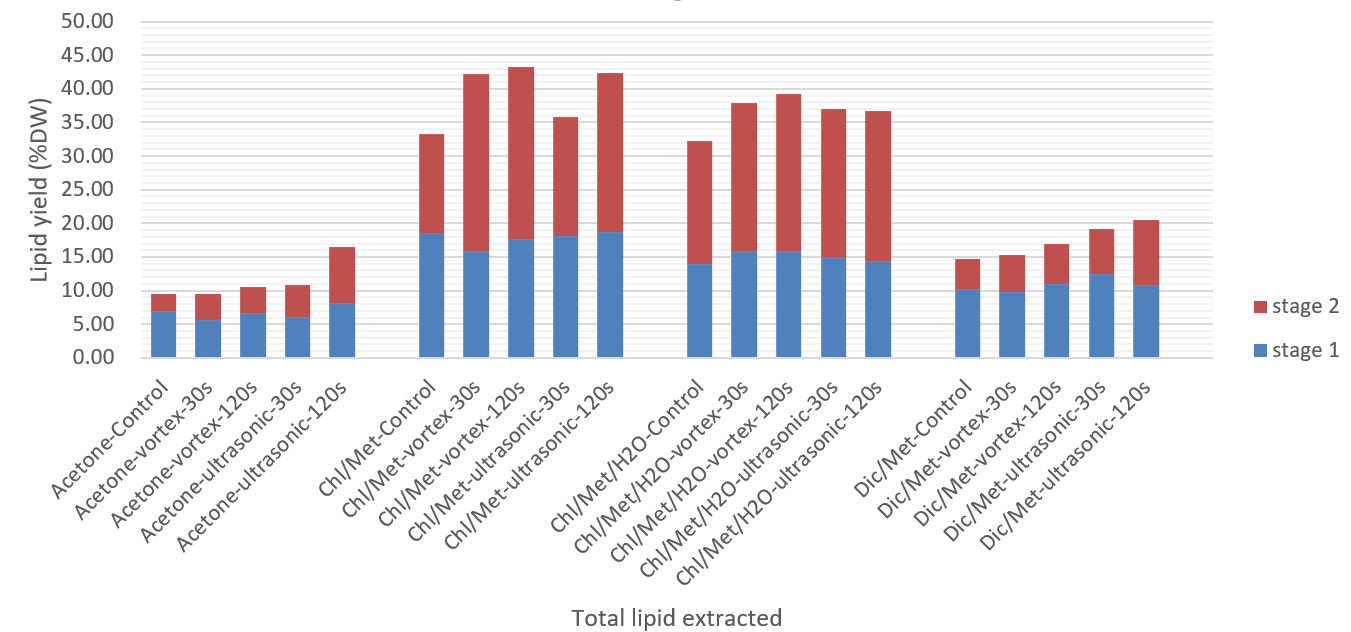


**Fig. S2 Replication-2 in *Phaeodactylum tricornutum***


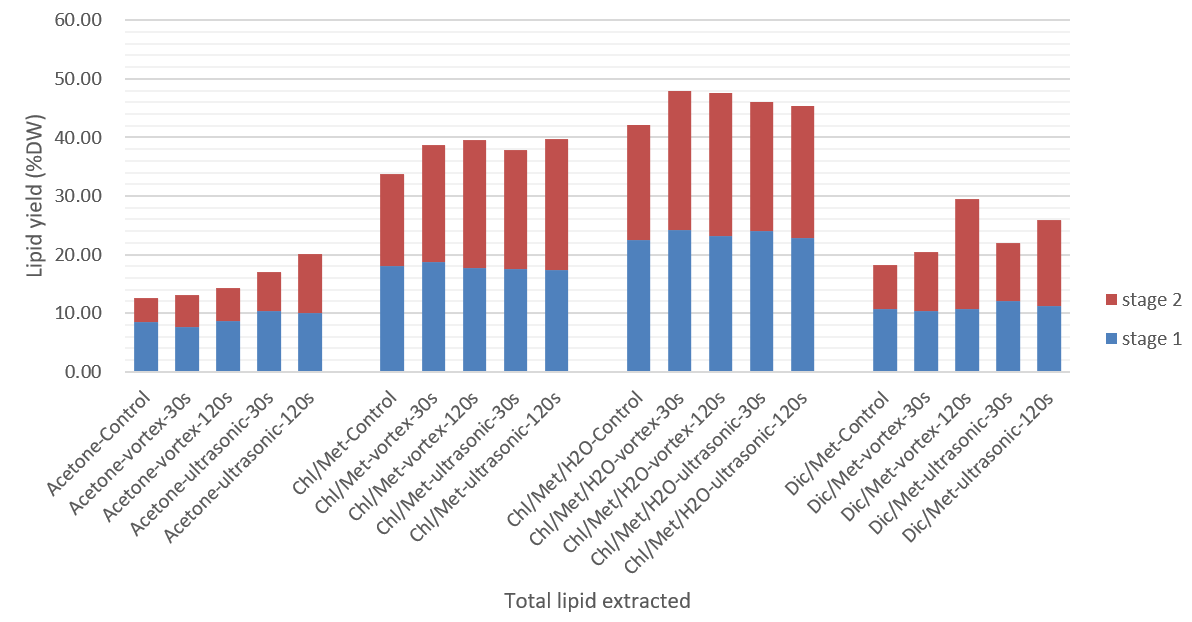


**Fig. S3 Replication-3 in *Phaeodactylum tricornutum***

**Fig. S4: Comparison the effects of vortex and ultrasonic water treatment on lipid extraction efficiency**

(Black bars represent the first-stage lipid extraction yield; grey bars represent the second-stage lipid extraction yield; Chl/Met represents chloroform/methanol extraction method; Chl/Met/H_2_O represents chloroform/methanol/H_2_O extraction method; Dic/Met represents dichloromethane/methanol extraction method)


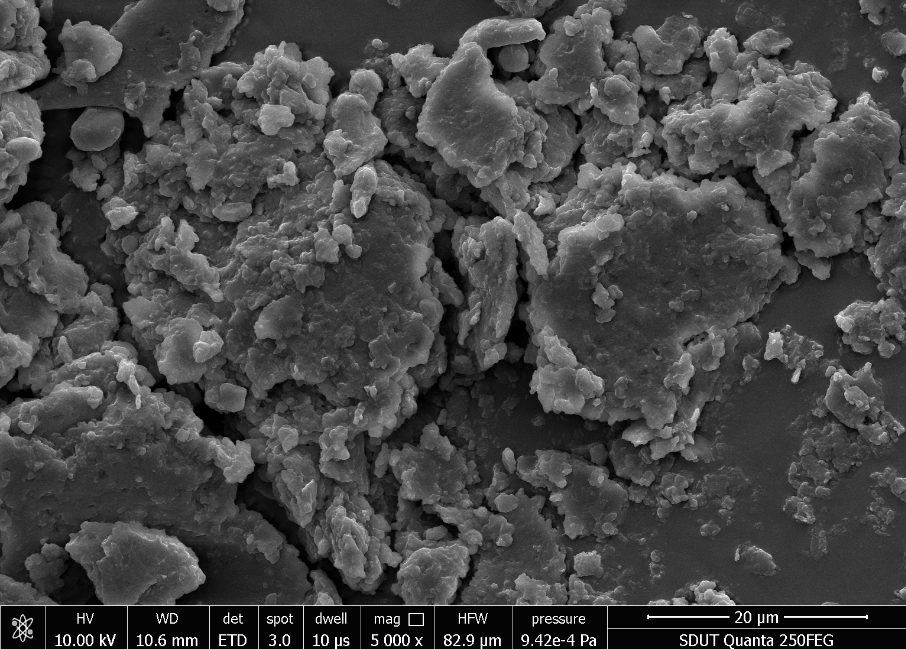


**（a）**


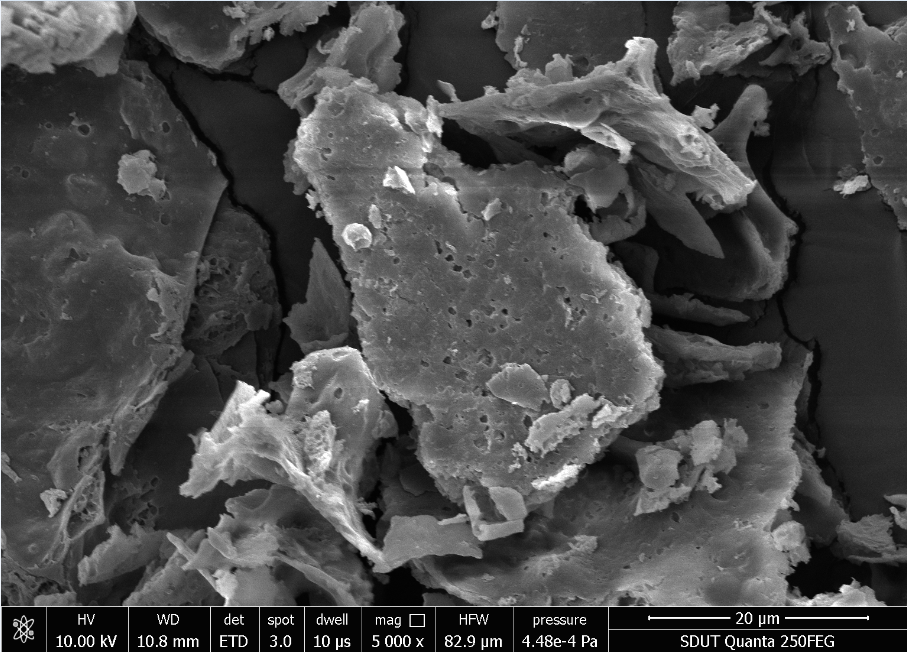

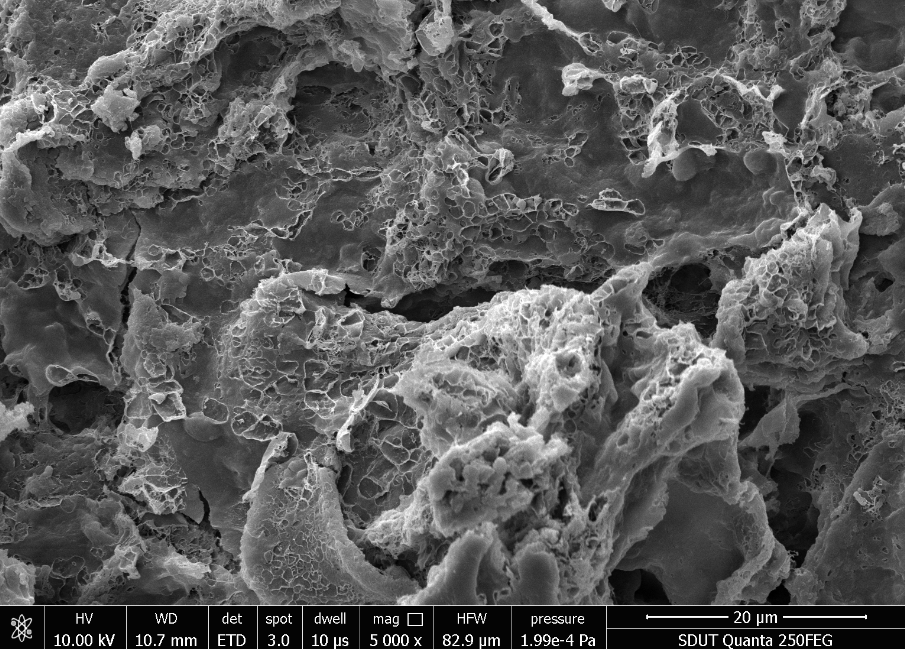

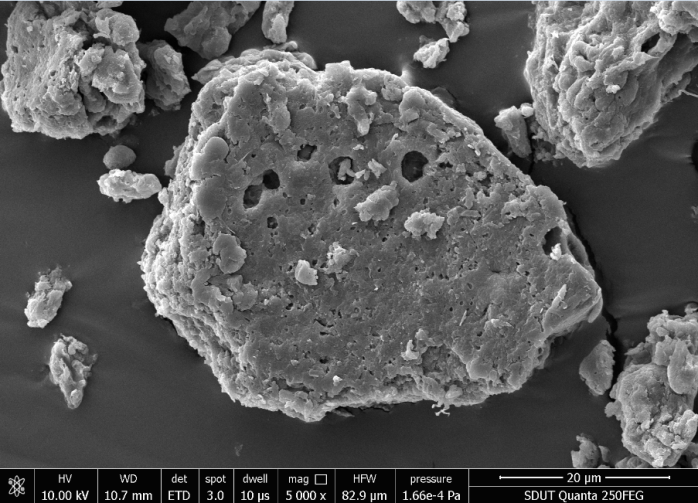


**（b）**

**（c）**

**（d）**

**Fig. S5. Scanning electron microscopy（SEM）of *Phaeodactylum tricornutum* cells in the organic solvent-water- organic solvent extraction process.**

**(a) Morphology of cells before extraction,**

**(b) morphology of cells after the first extraction,**

**(c) morphology of cells after water treatment**

**(d) morphology of cells after the second extraction**

**Table S1: Total extracted lipids (mg lipids/ gram cell dry weight)**

|  | Day3 | Day5 | Day9 |
| --- | --- | --- | --- |
| Acetone control | 83.67 | 95.15 | 125.47 |
| Acetone-vertex-30s | 89.10 | 95.39 | 147.69 |
| Acetone-vertex-120s | 124.32 | 105.23 | 132.30 |
| Acetone-ultrosonic-30s | 90.47 | 108.60 | 169.58 |
| Acetone-ultrosonic-120s | 97.33 | 165.26 | 201.01 |
|  |  |  |  |
| Chloroform/methanol-control | 201.07 | 333.06 | 337.94 |
| Chloroform/methanol-vertex-30s | 225.59 | 422.74 | 387.15 |
| Chloroform/methanol-vertex-120s | 258.78 | 432.70 | 395.96 |
| Chloroform/methanol-ultrosonic-30s | 256.26 | 357.77 | 378.18 |
| Chloroform/methanol-ultrosonic-120s | 272.93 | 422.99 | 397.85 |
|  |  |  |  |
| Chloroform/methanol/H2O-control | 259.71 | 321.85 | 420.41 |
| Chloroform/methanol/H2O-vertex-30s | 319.34 | 378.30 | 478.82 |
| Chloroform/methanol/H2O-vertex-120s | 358.40 | 392.01 | 475.76 |
| Chloroform/methanol/H2O-ultrosonic-30s | 310.29 | 369.54 | 460.53 |
| Chloroform/methanol/H2O-ultrosonic-120s | 326.97 | 367.63 | 452.82 |
|  |  |  |  |
| Dichloromethane/methanol | 118.57 | 147.71 | 182.76 |
| Dichloromethane/methanol-vertex-30s | 127.72 | 153.45 | 204.47 |
| Dichloromethane/methanol-vertex-120s | 127.61 | 169.82 | 294.49 |
| Dichloromethane/methanol-ultrosonic-30s | 141.05 | 192.29 | 220.29 |
| Dichloromethane/methanol-ultrosonic-120s | 166.56 | 205.45 | 259.16 |
